# The Topo-Speech sensory substitution system as a method of conveying spatial information to the blind and vision impaired

**DOI:** 10.3389/fnhum.2022.1058093

**Published:** 2023-01-26

**Authors:** Amber Maimon, Iddo Yehoshua Wald, Meshi Ben Oz, Sophie Codron, Ophir Netzer, Benedetta Heimler, Amir Amedi

**Affiliations:** ^1^Baruch Ivcher School of Psychology, The Baruch Ivcher Institute for Brain, Cognition, and Technology, Reichman University, Herzliya, Israel; ^2^The Ruth and Meir Rosenthal Brain Imaging Center, Reichman University, Herzliya, Israel; ^3^Gonda Brain Research Center, Bar Ilan University, Ramat Gan, Israel; ^4^Center of Advanced Technologies in Rehabilitation (CATR), Sheba Medical Center, Ramat Gan, Israel

**Keywords:** sensory substitution, spatial perception, sensory substitution device (SSD), blind and visually impaired people, sensory development, sensory perception

## Abstract

Humans, like most animals, integrate sensory input in the brain from different sensory modalities. Yet humans are distinct in their ability to grasp symbolic input, which is interpreted into a cognitive mental representation of the world. This representation merges with external sensory input, providing modality integration of a different sort. This study evaluates the Topo-Speech algorithm in the blind and visually impaired. The system provides spatial information about the external world by applying sensory substitution alongside symbolic representations in a manner that corresponds with the unique way our brains acquire and process information. This is done by conveying spatial information, customarily acquired through vision, through the auditory channel, in a combination of sensory (auditory) features and symbolic language (named/spoken) features. The Topo-Speech sweeps the visual scene or image and represents objects’ identity by employing naming in a spoken word and simultaneously conveying the objects’ location by mapping the x-axis of the visual scene or image to the time it is announced and the y-axis by mapping the location to the pitch of the voice. This proof of concept study primarily explores the practical applicability of this approach in 22 visually impaired and blind individuals. The findings showed that individuals from both populations could effectively interpret and use the algorithm after a single training session. The blind showed an accuracy of 74.45%, while the visually impaired had an average accuracy of 72.74%. These results are comparable to those of the sighted, as shown in previous research, with all participants above chance level. As such, we demonstrate practically how aspects of spatial information can be transmitted through non-visual channels. To complement the findings, we weigh in on debates concerning models of spatial knowledge (the persistent, cumulative, or convergent models) and the capacity for spatial representation in the blind. We suggest the present study’s findings support the convergence model and the scenario that posits the blind are capable of some aspects of spatial representation as depicted by the algorithm comparable to those of the sighted. Finally, we present possible future developments, implementations, and use cases for the system as an aid for the blind and visually impaired.

## 1. Introduction

Vision is commonly accepted to be the principal mediator between the objective world around us and the representation of what we perceptually experience (Cattaneo and Vecchi, 2011; Hutmacher, 2019). Visual input is known to be so dominant that it heavily influences the manner in which our other senses are processed (Posner et al., 1976), as is exhibited by well-known illusions such as the McGurk (McGurk and MacDonald, 1976) and ventriloquist effects (Bruns, 2019). Concerning spatial perception in particular, vision is considered to be especially important in forming spatial representations (Gori et al., 2014, 2020). Forming spatial representations involves acquiring a holistic image of objects and information concerning their distance, locations, and orientations relative to one’s self (Struiksma et al., 2009), information classically thought to be reliably acquired predominantly through vision and insufficiently conveyed through the other senses such as audition and touch (Battal et al., 2020). Despite this, vision and audition specifically are known to be the main routes for perceiving extra-personal space, with other senses, such as the tactile sense, being associated mainly with peri-personal space and the area surrounding one’s body (Van der Stoep et al., 2017).

The visually impaired and the blind gather information about their environments through multiple channels of the remaining senses. Philosopher Diderot’s letter on the blind for the use of those who can see depicts this as follows: “The man-born-blind of Puiseaux works out how close he is to the fire by how hot it is, how full a receptacle is by the sound liquid makes as he decants it, and how near he is to other bodies by the way the air feels on his face (Tunstall, 2011, p. 177).” Despite the general dominance of vision, the visually impaired and the blind are known to compensate for their lack of the sense of vision by utilization of the other senses (Röder et al., 2004; Bauer et al., 2017).

As far back as biblical times, the blind used canes, similar to what we now call “white canes,” as an aid in localizing and spatial orientation within their surroundings (Strong, 2009). However, not all of the blind population use a cane regularly (Blindness Statistics, n.d.). Furthermore, canes are nearly not employed at all to aid the visually impaired (Blindness Statistics, n.d.), who rely on a combination of their existing/residual vision.

A method employed by the blind specifically for acquiring spatial information is echolocation. Echolocation specifically allows for acquiring spatial representations in silent conditions, in contrast to relying on auditory cues for acquiring information from the surroundings. Echolocation, colloquially attributed to bats and dolphins in the wild, is also used by some of the blind population in a similar manner. Human echolocators make clicking sounds with their tongues and carefully listen to the echoes reverberating back to them from the objects in their surroundings. New technologies incorporate an element of color into echolocation-inspired devices, such as the EyeMusic (Abboud et al., 2014) and the Colorphone (Bizoń-Angov et al., 2021), which also incorporates a dimension of depth.

Neuroscientific findings indicate that blind expert echolocators activate the visual cortex when echolocating, specifically MT +, an area considered to be correlated with the perception of visual motion in the sighted (Thaler and Goodale, 2016). In addition, it has been shown that the sounds of echoes bouncing off of different objects activate the lateral occipital cortex, an area specifically related to shape processing, mainly through the visual perception of objects, but research conducted by our lab has shown that this area is multisensory in that it is also activated for shape processing when the information is conveyed through the tactile modality (Amedi et al., 2001, 2007). In both blind and sighted trained echolocators, a major factor underlying the ability to perform localization of objects using echolocation successfully is the element of pitch (Schenkman and Nilsson, 2011). As such, it can be understood that pitch is important for conveying spatial information. In the Topo-Speech algorithm, differences in pitch represent different locations on the y-axis, which may make the algorithm more intuitive, though this warrants future exploration as it is possible that pitch in echolocation might be helpful due to the reflection characteristics of the objects, while no such phenomena is explored here.

Braille reading and spoken language have also both been correlated with visual cortex activation (Sadato et al., 1996, 1998; Büchel et al., 1998; Seydell-Greenwald et al., 2021). The Braille reading method, invented in 1824, can be considered one of the earliest sensory substitution methods (Ptito et al., 2021). Braille conveys verbal information through haptic or tactile stimulation (Kristjánsson et al., 2016). Braille readers must use extreme accuracy and sensitivity to discriminate between patterns of raised dots with their fingers and translate this code into meaningful semantic information (Hamilton and Pascual-Leone, 1998). This indicates that language can serve as a potential substitute in the absence of vision.

It has been suggested (Röder et al., 2004) that when vision is unavailable, the following three scenarios are possible: The first scenario posits that there will be a lack or decrease in the sensory capabilities due to the lack of an essential sense (vision) (Gori et al., 2014; Cappagli et al., 2017b; Martolini et al., 2020a). The second is that no difference will be observed (Haber et al., 1993; Morrongiello et al., 1995). While the third suggests that there will be compensation, defined as better performance or surpassing the capabilities seen in the sighted, due to other sensory mechanisms making up for the lack of the visual sense (Roder et al., 1999; Amedi et al., 2003, [Bibr B6][Bibr B32]; Sabourin et al., 2022).

This is currently a central debate, with different bodies of evidence supporting the various hypotheses (Röder et al., 2004). In this study, we ask which of these scenarios is better supported. Moreover, we explore a specific kind of spatial perception induced by the Topo-Speech system in the visually impaired, a group underrepresented in the wealth of literature that mainly compares the sighted and the blind. We explored the difference (or lack thereof) in the performance of the visually impaired vs. the blind when using this system for conveying a certain kind of spatial perception. In addition, we explore the performance of the visually impaired population with the system as well. Exploring the abilities of the visually impaired in this case could serve as a particularly interesting intermediate in that their sensory development with respect to vision is distinct from that of both the sighted and the blind.

Recently we developed a novel sensory substitution algorithm in our lab that combines verbal and spatial information (Heimler et al., 2019; Netzer et al., 2021). The Topo-Speech algorithm used in the current study represents the verbal naming of an object in a way that conveys its location in space such that in the vertical axis, objects located higher are represented by a higher pitch and lower by a lower pitch. The horizontal axis is mapped temporally from left to right, such that the closer the object is to the left, the sooner one hears the stimulus. This representation provides the user with information that allows them to simultaneously know both the identity of the objects and their locations in space by correlating the spoken word (for identity) with defined auditory characteristics (for location).

Prior research has shown that sighted individuals can successfully learn to use this algorithm for identifying spatial positions after undergoing a single training session and even generalize to locations they had not been trained on (Netzer et al., 2021). Yet thus far, research exploring this system has provided a first proof of concept focused on the technical aspects of the Topo-Speech algorithm and the ability of sighted blindfolded participants. This study on the sighted showed that they were able to understand and use this system with success levels well above chance level. This study expands upon these findings to explore the applicability of this approach, and its advantages and disadvantages, in visually impaired and blind individuals.

The key, primary, goal of this present study is practical—to extend the prior research to assess the ability of the visually impaired and blind to understand the algorithm and explore whether their modified visual experience throughout life influenced their ability to perform with the system. The study aims to provide a proof of concept for the possible future development of the algorithm as an aid for the visually impaired. In addition, we demonstrate how some aspects of spatial information can be transmitted through non-visual channels. More specifically, the correlation between a sensory method for conveying spatial information through audition, and a symbolic one, for conveying object identity through language. We suggest that this is of particular significance because it allows for taking the high-complexity visual data and translating it to symbolic representation, alongside lower bandwidth data to a sensory representation.

Another complementary goal of this study is more theoretical. While there is very little dispute concerning the dominance of vision in sensory perception and in forming our holistic representation of the world (Cattaneo and Vecchi, 2011; Cappagli and Gori, 2019; Hutmacher, 2019), we know that the human brain (as the root of how we perceive the world around us) is exceedingly capable of adaptation to its circumstances and forthcomings.

These matters are pertinent due to the differential neurodevelopment in the visually impaired/blind as compared to the sighted and the distinct experiences of the different populations with respect to forming spatial representations throughout their lives. This is relevant for another debate of whether the deficit (insofar as there is one) concerning spatial perception is indeed perceptual or cognitive (Bleau et al., 2022). For example, it is known that the human brain is structured such that it can compensate by way of other senses. There are two main strategies for this, one it the “taking over” of visual function by an increase in the efficiency of other functions (for a review, see Bedny, 2017), and the other is through sensory substitution (for a review, see Maidenbaum et al., 2014a). Sensory substitution is the transfer of information commonly provided through one sense through an alternate sense. The sensory substitution method used in the current study uses a sweep line technique, whose use in sensory substitution owes its beginning to the vOICe (Amedi and Meijer, 2005) sensory substitution device (SSD) that introduced an algorithm that scans the visual scene from left to right. It translates it into sounds using spectrographic sound synthesis and other audio enhancement techniques, pixel by pixel. The corresponding series of sounds is known as a “soundscape,” in which the horizontal axis is represented by the time of presentation and panning, while the vertical axis corresponds to tone frequency, and the level of intensity (loudness) of the sound represents brightness (Striem-Amit et al., 2012). Other SSDs employing the sweep line technique incorporate additional dimensions of the visual scene in the transformation, such as the EyeMusic, which represents a color dimension through different timbres of sound in a musical pentatonic scale (Abboud et al., 2014). We will inquire into the question of which mechanism is taking place by employing the method of sensory substitution of spatial information commonly acquired by vision in the sighted through auditory properties.

Yet another powerful mediator between the world and our perception of it is language. Throughout the history of mankind, spoken language has served as a distinguishing feature of humans from other species. Language is considered so powerful that it was thought to threaten god’s supremacy in the story of the tower of Babel. As such, it is no surprise that our brains are very much attuned to language processing. In the brain, language plays such a significant role that the visual deprivation in the blind sparks neuroplastic mechanisms which enable higher cognitive functions, such as language processing, to activate the visual cortex (Amedi et al., 2004; Merabet et al., 2005; Bedny, 2017) alongside specific spatial language processing (Struiksma et al., 2011).

Furthermore, research indicates that language provides a central means for acquiring spatial information in the blind (Afonso et al., 2010). Though symbolic and not sensory, language has been shown to bring about spatial representations to the same extent as perceptual auditory information (Loomis et al., 2002). Following these insights concerning the significance of language, our lab has previously developed the Topo-Speech algorithm that conveys, *via* spoken language, object identity *via* spoken language (Heimler et al., 2019; Netzer et al., 2021). As such, the current study is not only practical, but we lay the groundwork for further research exploring the perception of space *via* language and sensory information of those with no visual experience.

## 2. Materials and methods

### 2.1. Participants

Twenty two adults (10 Female) participated in the study, eight of whom are blind and 14 visually impaired (Table 1). Blind participants in the study were determined *via* a certificate of blindness. A person is entitled to receive the certificate if they are totally legally blind or with visual acuity of 3/60 m (Ministry of welfare and social security, n.d.). The visually impaired do not have a blind certificate, so their status as visually impaired was verified according to their entitlement to obtaining a driving license. As defined by national regulation, to be eligible for a driver’s license, one must present with visual acuity between 6/12 and 6/6 m (Assuta-optic, n.d.). Those who do not have visual acuity in this range, even after vision correction methods such as glasses, are defined as visually impaired. Visually impaired participants were blindfolded during the experiment. When speaking of the sighted, we are comparing to the participants in Netzer et al. (2021). Their participants were 15 sighted adults (nine women; aged 27.2 ± 1.57 years). All participants had no known hearing/balance impairments or neurological conditions. All participants were above the age of 18 with a mean age of (35.64 years ± 10.75 years). None of the participants had prior experience with either the Topo-Speech algorithm nor any other sensory substitution device. This study received full ethics approval from the Reichman University Institutional Review Board (IRB). All participants received monetary compensation of 80 shekels per hour for their participation in the experiment alongside reimbursement for their transportation to and from the university.

**TABLE 1:** Participant information.

#	Age	Visual impairment	Details
1	35	Vision impaired	Retinopathy of prematurity, nystagmus
2	41	Vision impaired	Retinitis pigmentosa
3	59	Late blind	Retinitis pigmentosa, glaucoma, blind since age 44
4	22	Vision impaired	Glaucoma
5	57	Vision impaired	Retinitis pigmentosa
6	29	Early blind	Glaucoma, Blind since age 15
7	29	Congenitally blind	
8	38	Congenitally blind	
9	31	Congenitally blind	
10	37	Vision impaired	Retinitis pigmentosa
11	38	Congenitally blind	
12	26	Vision impaired	Photophobia
13	43	Vision impaired	Stickler syndrome
14	44	Vision impaired	Optic atrophy
15	28	Vision impaired	Albinism
16	26	Vision impaired	Septo-optic dysplasia/de-morsier
17	24	Vision impaired	Retinal degeneration
18	52	Vision impaired	Cataract, nystagmus
19	25	Vision impaired	Albinism, astigmatism, nystagmus
20	42	Vision impaired	Cone dystrophy
21	23	Congenitally blind	
22	35	Congenitally blind	

### 2.2. Experiment design and algorithm

The Topo-Speech algorithm is a sweep line algorithm that scans the visual scene from left to right. The x axis of the visual scene or image is mapped to time and represents the objects’ horizontal locations, while the y-axis of the visual scene or image is mapped to the pitch of the soundscape. As such, the algorithm functions in a manner such that if one hears word 1 followed temporally by word 2, then corresponding object 1 was located further to the left of the visual scene than corresponding object 2 (representing the x axis). If one hears word 1 higher in pitch than word 2, then corresponding object 1 is located higher in the visual scene than corresponding object 2. The content of the words represents the identity of the object scanned (for example shoe, book). For the purpose of this study, a database of 60 highly frequent words in the Hebrew language was professionally recorded, after which the words were modified for the format of the Topo-Speech algorithm and trials using the Audacity audio editing software. The training stage consisted of 27 trials in total. During training, trials were presented in a random order, where each of the nine possible locations was tested three times, and a word could not appear twice in the same location. The testing stage consisted of 90 trials in total, with words appearing in each location 10 times. The words used were all matched to two syllables for consistency and represented objects with no inherent spatial content, such as “na-al” (shoe) and “se-fer” (book) instead of “sky” and “carpet” which may be associated with upper and lower parts of space, respectively. See Table 2 for the complete list of words. Two short consecutive beeps signified each word presentation’s start and end points.

**TABLE 2:** Words used in the experiment.

English translation	English transliteration	Word in Hebrew
**Words used in the training stage of the experiment**		
Earring	Agil	עגיל
Window	Chalon	חלון
Wand	Sharvit	שרביט
Flower	Perah	פרח
Hat	Kova	כובע
Chair	Kise	כיסא
Newspaper	Iton	עיתון
Doll	Buba	בובה
Flag	Degel	דגל
Carrot	Gezer	גזר
Box	Argaz	ארגז
Branch	Anaf	ענף
Pitcher	Kankan	קנקן
Shoe	Naal	נעל
Balloon	Balon	בלון
Pot	Atzitz	עציץ
Soap	Sabon	סבון
Sock	Gerev	גרב
Boots	Magaf	מגף
**Words used in the testing stage of the experiment**		
Mug	Sefel	ספל
Ruler	Sargel	סרגל
Lighter	Matzit	מצית
Wallet	Arnak	ארנק
Bottle	Bakbuk	בקבוק
Ball	Kadur	כדור
Hammer	Patish	פטיש
Knife	Sakin	סכין
Notepad	Pincas	פנקס
Shirt	Chultza	חולצה
Computer	Machshev	מחשב
Flute	Chalil	חליל
Lemon	Limon	לימון
Fork	Mazleg	מזלג
Teaspoon	Capit	כפית
Orange	Tapuz	תפוז
Kettle	Kumkum	קומקום
Glove	K’fafa	כפפה
Leaf	Aleh	עלה
Binder	Klasser	קלסר
Box	Kufsa	קופסה
Lock	Manul	מנעול
Cable	Chevel	חבל
Bobby Pin	Sika	סיכה
Stapler	Shadchan	שדכן
Pan	Machvat	מחבת
Letter	Michtav	מכתב
Paintbrush	Mik’hol	מכחול
Button	Kaftor	מכחול

A 3 × 3 grid was created for the experiment, with the dimensions 80 × 80 cm. The grid was hung on the wall at 160 cm. A digital beep indicated the beginning of the word presentation, followed by a word stating the object’s identity presented after various delays from the initial beep. A second beep indicated the end of the word presentation, thus defining the borders of the x-axis. The spatial location of each word could be presented in one of 3 different pitches in the y-axis: Pitches Low C, Low A#, and Middle G#, and each trial lasted a total of 2 s.

Before beginning the training, participants received a general explanation about the concept of sensory substitution devices: “Sensory substitution devices (SSDs) are algorithms that convey visual information *via* other sensory modalities, in this case, audition. The algorithm does this substitution based on two principles: First, the algorithm uses language to identify the objects, and second, it uses their location in space.”

The participants were instructed to reach their hands forward and freely feel the grid. Meanwhile, they were told that the algorithm maps a space of three rows and three columns, creating a 3 × 3 grid. Then, participants were explained the rules by which they would locate the objects during the experiment as follows: “The X-axis is mapped to the time domain (from left to right). The participants hear a beep at the beginning and end of each word (and there is a difference between them so that you know when it is the beginning and when it is the end). The closer a stimulus is heard to one of the beeps, the participant will be able to perceive its proximity to the left or right ends of the grid. The y-axis is mapped to the pitch, so the higher the participant hears a word, the higher the corresponding stimulus will be placed on the grid.”

Participants underwent two stages during the study, a training stage and then a testing stage. They were asked to listen carefully to the auditory stimuli, after which they were instructed to touch the cell on the 3 × 3 grid that they thought represented the location of the object presented through audition. The participants touching of the grid represents sensorimotor compatibility to show that the auditory information can become spatial, and connects to the field with the help of the sensorimotor action of reaching out hands. Sending the hand to the location indicates that the person can perform this conversion. From an applicative point of view, the purpose of the algorithm is to help the blind and visually impaired to operate in the world, and when we are in the environment we don’t call out the names of objects to get them. Rather, we want the algorithm to tell us where the object is in space so that we can easily reach out and take it. Thus, the feedback is motor and not verbal.

During training the participants would listen to each word presentation, and receive feedback on their response with regard to the spatial location represented. They would receive feedback on whether their response was correct or incorrect, and if their answer was incorrect they were directed to the location of the correct answer in each training trial. They could repeat the playback of the word presentation as many times as desired, and if the response was incorrect, the same word was repeated. The training stage lasted for an average of 15:36 ± 9.06 min [mean ± standard deviation (SD)], with 14 min for visually impaired and 17 min for the blind. During the testing stage, consisting of 90 trials, each word was presented twice, after which a choice was made by the participant. No feedback was provided during this stage. The participant responses were recorded. Following every 30 trials, the participant was offered a 2-min break, which they could choose to not take if they wished. With regards to removing the blindfold during the break, visually impaired participants were turned around so as not to see the grid. The testing stage lasted for an average of 23:27 ± 5:34 min (mean ± *SD*).

**FIGURE 1:**
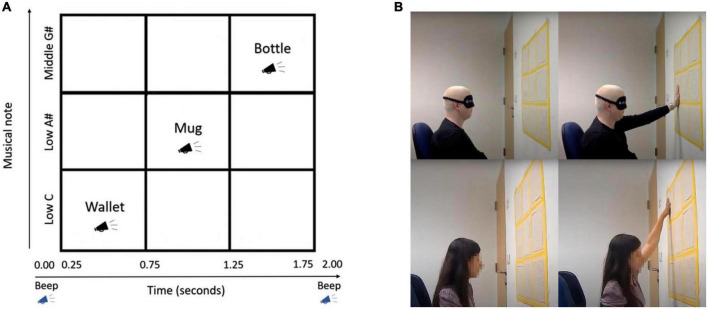
**(A)** Illustration of the Topo-Speech algorithm as employed in the current study (adapted from Netzer et al., 2021) **(B)** Experimental set-up for visually impaired (top) and blind (bottom).

Figure 1 illustrates the experimental set-up, the 3 × 3 grid, and depicts how the participants chose their answer during the experiment. The top two images are of a blindfolded visually impaired participant, the bottom two are images of a blind participant.

### 2.3. Statistical analyses

**FIGURE 2:**
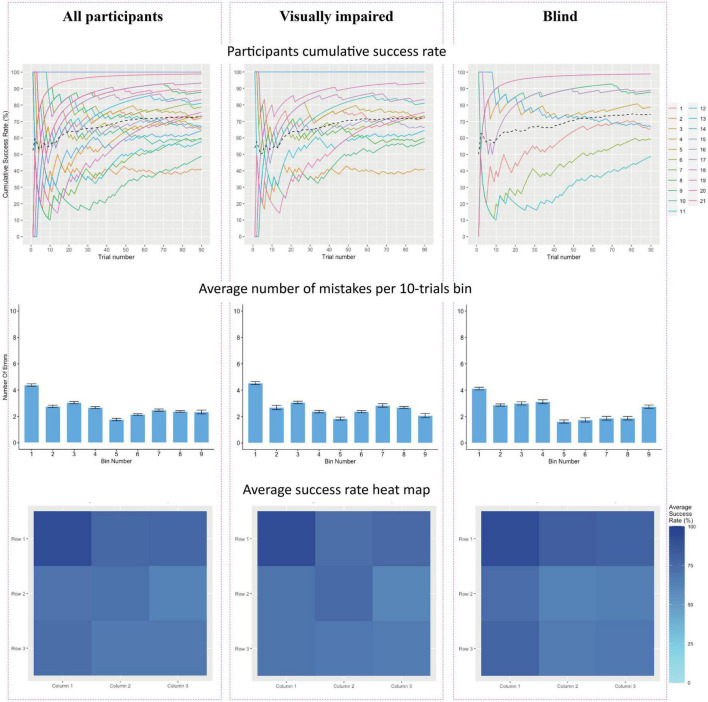
**(Top row)**- Cumulative success rates by trial number. Each participant is represented by a colored line, with the group average represented by a black dashed line. **(Middle row)** The average number of mistakes per 10 trials. **(Bottom row)** of figures portrays a heat map of the average success rate. Darker shades represent higher rates of success. **(Left column)** results across all participants (*n* = 21). **(Middle column)** Average success rates for blind participants (*n* = 8). **(Right column)** Average success rates for visually impaired participants (*n* = 13).

Because the sample sizes in both groups were relatively small (less than 50 participants), non-parametric statistical tests were used, including the Mann–Whitney to compare success rates between groups and the Wilcoxon signed-rank test (equivalent to the *t*-test) for independent samples comparisons, specifically the participants’ performance to chance level. These tests were performed using SPSS Statistics 25. Cumulative success rates and the average number of mistakes per 10 trials were calculated and shown below in Figure 2. The cumulative success rate is a variable calculated for each subject. For each subject, instead of calculating all of the successes on all the stages of the test at once, each time, a cumulative success rate is considered up to the current trial in order to assess the learning curve of the subjects. As such, we calculate the cumulative percentage of success the participant had up to a certain trial and divide it by the trial number.

## 3. Results

### 3.1. Both groups successfully learned to use the algorithm following a short training period

The group average and SD during the experiment were: 73.39% ± 15.89%. One visually impaired participant was removed due to his being extremely uncooperative during the experimental procedure. Table 3 specifies each participant’s success rates and standard deviation during the testing stage. Table 4 summarizes the success rates and SD of the individual participants divided into blind and visually impaired. The performance of all participants was greater than chance.

**TABLE 3:** Individual success rate and SD for each participant.

Subject number	Success rate (%)	SD (%)
1	72.22	45.04
2	41.11	49.48
3	65.56	47.78
4	73.33	44.47
5	73.33	44.47
6	78.89	41.04
7	58.89	49.48
8	87.78	32.94
9	48.89	50.27
10	60.00	49.26
11	66.67	47.40
12	57.78	49.67
13	82.22	38.45
14	64.44	48.14
15	100.00	0.00
16	66.67	47.40
17	83.33	37.48
18	95.56	20.72
19	75.56	43.22
20	90.00	30.17
21	98.89	10.54

**TABLE 4:** Summary of success rates of each group.

Impairment	Number of subjects	Success rate (%): mean (*SD*)
Blind	8	74.45 (± 17.20)
Visually impaired	13	72.74 (± 15.72)

**FIGURE 3:**
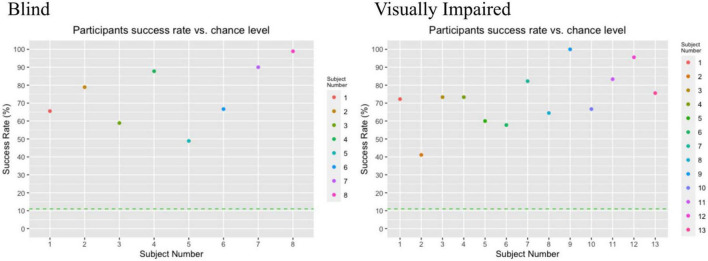
Individual participant success rate vs. chance level. Each subject is represented by a filled dot, and the chance level is represented by a dotted line, at position 11.00 on the y axis. The chance level is 11% success rate due to a correct answer being one of nine possible locations.

Figure 3 specifies the success rate of each individual participant compared to the chance level at 11%. As can be observed in the figure, all participants performed above chance level. This was confirmed with a one-sample Wilcoxon signed-rank test, indicating that the participants performed significantly better than chance level; *T* = 231.00, *z* = 4.015, *p* < 0.001.

### 3.2. The performance of the blind was not significantly different from that of the visually impaired

A Mann-Whitney test indicated no significant differences between group success rates, *U(N*_blind_ = 8, *N*_visually_ _impaired_ = 13) = 49.500, *z* = –0.181, *p* = 0.860.

### 3.3. The performance of the visually impaired and the blind was not significantly different from that of the sighted

A Mann-Whitney test revealed no significant difference between success rates in the present sample compared to a previous study that used sighted participants (Netzer et al., 2021); *U(N*_Non–sighted_ = 21, *N*_sighted_ = 14) = 101.00, *z* = –1.550, *p* = 0.127.

In order to analyze whether participants’ performance improved throughout the experiment, cumulative success rates for each participant were modeled. The top row of Figure 2 represents the result for each participant across all 90 trials, along with the group’s average cumulative success rate. All three graphs show a slight positive gradient, indicating improvement in success rate across the experiment, with a plateau from trial 50 onward. Another method to model participants’ learning during the experiment is by assessing the number of mistakes made by participants during the experiment. This is depicted in the middle row. The experiment was divided into nine bins of ten trials each, in which the average number of participant errors was calculated for every 10 trials.

To represent the success rates across participants, heat maps were created, where the average success rate for each square on the 3 × 3 grid is represented with a cell in the graph. The first graph on the bottom row highlights that while all participants had very high success rates, they identified the top-left cell with the highest accuracy and were least accurate in identifying the middle-right cell. The bottom middle and bottom right graphs present heat maps of blind and visually impaired participants separately. Visually impaired participants identified the top left the most accurately, along with top-right and middle closely after, whereas blind participants revealed a clear advantage in identifying the top row, as well as on the left, with top-left and bottom-left having the highest success rate.

## 4. Discussion

Prior research conducted in our lab showed that sighted blindfolded participants could learn to use the Topo-Speech algorithm with an accuracy of 80.24 percent (Netzer et al., 2021). In this study, we expanded upon this by evaluating for the first time this system’s ability to convey spatial information to the population it was aimed at: the blind and visually impaired. The current study results show that both the blind and visually impaired were capable of learning to use the algorithm with success rates comparable to those of the sighted, with no statistically significant difference in their performance. The blind showed an accuracy of 74.45 percent, while the visually impaired had an average accuracy of 72.74. It is apparent that the blind performed better than the visually impaired, though not significantly better. The same trend held for training, with the blind having a shorter training time than the visually impaired but a longer training time than that shown in the (blindfolded) sighted participants in our previous study, though the difference was not found to be statistically significant (averaging ∼10 min for sighted, 14 min for visually impaired and 17 min for the blind). As such, the main goal of this study—to test whether the system is highly intuitive also to fully blind or visually impaired—has been positively achieved. In this study, we were primarily interested in assessing the system’s feasibility and practical functionality for the blind and visually impaired. As such, a limitation to be noted concerning the comparisons to the sighted is that the groups were not matched for age and gender. Future studies could perform a direct comparison between these groups as well.

### 4.1. Spatial perception and representation when vision is lacking

Concerning the more theoretical debate—a central debate with relation to blindness, congenital blindness, in particular, is the ability or inability of the blind to reach the same or a similar level of skill as the sighted with respect to numerous tasks. This question is particularly interesting with relation to tasks in which vision is classically thought to play a central role, for example, spatial perception. On the one hand, it was commonly accepted that the blind and visually impaired have a significant impairment with respect to their sense of space and capability for forming spatial representations (Gori et al., 2014; Cappagli et al., 2017b; Martolini et al., 2020a). However, research now indicates that following dedicated training, the blind can become more capable of spatial localization (Gaunet et al., 1997; Cappagli et al., 2017a). It is now thought that the blind can perform spatial tasks with the same level of ability as the sighted when the information is delivered as auditory or tactile input, and some research even indicates that they may reach better performance (Roder et al., 1999; Collignon et al., 2006). In addition, three lifelong models represent trajectories of obtaining a spatial understanding in the blind. Two models suggest that vision is such a crucial element in spatial knowledge that the blind devoid of vision, are at an insurmountable disadvantage. The persistent model states that the blind have an initial disadvantage compared to the sighted, which persists throughout life. The cumulative model posits that the disability not only persists but even leads to an increase in the discrepancy between the abilities of the blind and the sighted (the blind improve in their abilities over time while the blind do not). The convergent model, on the other hand, suggests that the blind have an initial disadvantage with respect to spatial knowledge, and yet this “converges” with the abilities of the sighted as a result of experience throughout life and training, whether explicit or implicit (Schinazi et al., 2016; Aggius-Vella et al., 2017; Finocchietti et al., 2017; Cuppone et al., 2018, 2019; Cappagli et al., 2019; Martolini et al., 2020b,^2022^). It is now known that the blind brain provides for compensatory mechanisms for lack of vision and visual deprivation from numerous studies showing activation of the visual cortex in response to various spatial tasks (Striem-Amit et al., 2012; Abboud et al., 2015 and also reviews by Kupers and Ptito, 2014; Maidenbaum et al., 2014a; Ricciardi et al., 2014). The findings of this study support the convergence model alongside the scenario that posits that the ability of the blind and the visually impaired to understand a subset of spatial representation delivered by the auditory information provided by the system is not inferior. Moreover, the wealth of research showing improved performance in the blind population after training corresponds to this direction of the convergence model, therefore agreeing with and supporting our hypotheses.

The current study also strengthens research indicating that while the blind are capable of some aspects of spatial perception to a similar extent as the sighted, the visually impaired show (a non-significant in our case) trend for slightly poorer performance. A previous study showed that individuals with low residual vision (peripheral) were less capable of sound localization and performed worse than both their blind and sighted counterparts (Lessard et al., 1998). Another study showed that children with visual impairments are less capable of updating spatial coordinates as compared to the sighted (Martolini et al., 2020a). It could be that in the case of the visually impaired, compensatory neuroplasticity takes place to a lesser extent than in the blind, and yet their vision is severely impaired in comparison to the sighted. On the other hand, Cappagli et al. (2017b) found that while the blind perform more poorly on tasks related to spatial hearing, the visually impaired perform at the same level as their normally sighted peers.

In this study, alongside the objective qualitative experiment, the participants were also asked several subjective questions related to spatial perception and their user experience with the training and algorithm during the experiment. Their responses, though anecdotal, emphasize some interesting points. When asked, “How do you get information about the locations of objects in your environment in everyday life?” The visually impaired showed an automatic tendency to lean on residual vision, even when it is limited in capacity, and other senses are fully functional and intact. This strengthens the hegemony of the visual modality with respect to spatial perception, as all but one of the visually impaired indicated vision as their main mode of acquiring a spatial representation of objects in their surroundings. For example, one participant replied, “My vision is good enough for me to manage without relying on my hearing.” Another stated, “I see, and when I can’t, then I use hearing.” These subjective reports support the interpretation expressed by Cattaneo and Vecchi (2011), who ask, “why is vision so important in our life?” The answer is quite pragmatic: because the visual is “easy.” On the other hand, all but two of the blind participants, who cannot default to vision, reported not only using hearing, but many specifically reported that they rely on “asking” other people, thereby acquiring the information through language. One participant said: “If it’s an environment I know, I know everything where it is, and if I’m in a new environment, I don’t know it, then I have to ask and study it first and then I move on” and another responded: “By asking people or arranging the objects in an order that I can choose.” This comes alongside hearing, as expressed by another participant: “According to the sound mainly and later I make a map in the head of the structure and everything.” These reports further support the understanding that due to their reliance on their (limited) visual capabilities as opposed to other wholly intact sensory modalities, compensatory neuroplasticity is less likely to take place, and if it does, to a lesser degree than in the blind. Even more so, it is likely that their defaulting to vision has a detrimental effect on their ability to compensate behaviorally by way of other senses, as is exhibited by the Colavita effect (Colavita, 1974; Spence et al., 2011).

### 4.2. Conveying spatial information through non-visual channels

While our prior research provided a proof of concept for the general usability of the algorithm as a method of conveying spatial information through language, the current findings take this one step forward. We believe this study serves as a proof of concept for using language-based sensory substitution systems such as the Topo-Speech to aid the visually impaired and the blind.

Some tools developed for the blind and visually impaired tackle the issue of spatial localization from the practical perspective, designed, for example, to allow the blind to gather information from their surroundings specifically pertaining to distances, navigation, and obstacle detection for a particular aim, such as independent mobility. One such tool, the EyeCane, is an electronic travel aid (ETA) that relies on multiple sensory stimuli. The EyeCane, for example, integrates auditory cues with haptic ones allowing the user to identify objects and barriers in their surroundings by manipulations in the frequency of the multisensory cues (Chebat et al., 2011, 2015; Buchs et al., 2017; Maidenbaum et al., 2014b). For an extensive review of other technologies developed for assisting the blind, see Ptito et al. (2021). A particularly interesting avenue for further research with this algorithm related to the integration of haptic and auditory cues could be the association between Braille reading (or lack thereof) and success with using the Topo-Speech algorithm. All of the blind participants in this study were Braille readers.

Considering these findings, we speculate that Braille readers may have higher success rates and find the Topo-Speech algorithm more intuitive than non-Braille readers. This is further supported by the correspondence between Braille reading, which is from left to right, and the Topo-Speech in which a word presented temporally closer to the beginning of the stimulus presentation is closer to the left side of the “visual field.”

Braille reading also conveys language information through haptic, or tactile, stimulation (Kristjánsson et al., 2016), as Braille readers must use extreme accuracy and sensitivity to discriminate between patterns of raised dots with their fingers and translate this code into meaningful semantic information. Various studies have shown that Braille readers have an enlarged sensory representation of the reading finger, compared to sighted and blind non-Braille-readers, through recording somatosensory evoked potentials (Pascual-Leone and Torres, 1993). Mapping the motor cortical areas that represent reading fingers through transcranial magnetic stimulation has revealed that this enlargement is also seen in blind Braille readers (Pascual-Leone et al., 1993). A plausible explanation of these research findings is that the afferent information extracted by blind Braille readers from their fingerpad may be more detailed and specific, causing them to succeed in the discriminatory task that is Braille reading (Hamilton and Pascual-Leone, 1998).

Braille reading also connects to another interesting finding of this study. Compared to the visually impaired and the sighted, the blind participants showed a clearer tendency to correct answers on the left side of the answer grid. Research indicates that spatial orientation and processing is lateralized to the right hemisphere in the blind (Rinaldi et al., 2020) as well as in the sighted (Vogel et al., 2003). This right hemisphere lateralization has been shown to be more prominent and substantial in the blind (Rinaldi et al., 2020). One of the mechanisms correlated with this is Braille, written from left to right (Rinaldi et al., 2020) (similar to the stimuli presented in the present experiment: a word closer to the first beep occurs on the left side of the visual field or closer to zero on the X-axis). Nevertheless, this warrants further research, as an alternative explanation for this could be more trivially related to the ability to perceive time exactly. This could be tested by exploring a change in the direction of the sweep.

Braille represents a language system composed of symbols, the Braille letters. The current study demonstrates the potential of using mixed methods for conveying information through sensory substitution coupled with symbolic means. Such mixed representations of information would use sensory substitution, in which the information is conveyed perceptually, alongside symbolic information, representing more complex combinations of information. In this case, location properties *via* audio features and spoken language that provide symbols our brains are attuned to processing. Training with purely visual to auditory sensory substitution devices (such as the EyeMusic upon which the Topo-Speech algorithm is based) can take tens of hours of training. On the other hand, the Topo-Speech algorithm can successfully be trained in short training sessions of under 20 min in all populations tested. We suggest that this is partly due to the combination as it allows for taking the high-complexity visual data and translating it to a symbolic representation and the scalable, lower bandwidth data to a sensory representation. This also serves to strengthen the interpretation of the brain as a task-selective, sensory independent organ. Under this interpretation, different brain areas are correlated with tasks (such as perceiving the 3D shape of objects) rather than senses (such as vision).

### 4.3. Future directions and implementations

To further refine and establish the findings of this work, we find it valuable to explore several adaptations of the algorithm and evaluation method. Particularly, we would wish to experiment with changing the direction of the sweep, the mapping of pitch ranges to height, and different time intervals. Such experiments could eliminate any biases caused due to the specific choice of one of these factors, as well as help in understanding their role in changing perception. Hence guiding future work in optimizing such representation algorithms.

With regard to expanding on the experimental design, a direction that we have previously explored in the sighted and could provide another meaningful evaluation of the blind and visually impaired would be to extend the spatial representation provided by the algorithm to the backward space. While vision in the sighted has a limited range of 210 horizontal degrees (Strasburger and Pöppel, 2002), audition spans the full 360 degrees. As indicated by our prior research with the Eyemusic algorithm (Shvadron et al., under review) and the Topo-Speech (Heimler et al., 2019; Netzer et al., 2021), it is possible to convey information from the back spatial field by way of audition. Our future implementation of the Topo-Speech algorithm could explore the feasibility and implications of such expansion of the spatial field in the blind and visually impaired, who are not susceptible to a limited range of vision in the front space to begin with.

Another possible adaptation of the Topo-Speech algorithm could incorporate a tactile element as well. We have previously shown how coupling tactile information to auditory speech using a “speech to touch” SSD enhances auditory speech comprehension (Cieśla et al., 2019, 2022). Adding this sensory modality to the algorithm may enhance its effectiveness by means of coupling tactile feedback to the auditory stimuli or adding more information, such as the representation of another dimension, such as depth *via* vibration intensity or frequency. This is further supported by an abundant body of research indicating that the blind show similar to better performance than the sighted, particularly in auditory and tactile tasks (Lessard et al., 1998; Van Boven et al., 2000; Gougoux et al., 2004, 2005; Voss et al., 2004; Doucet et al., 2005; Collignon et al., 2008).

Going forward, we aim to expand the capabilities of the existing algorithm beyond its current limitations by accounting for the lack of ability to represent objects simultaneously, offering a more continuous representation of the space, as well as more dimensional information describing a scene. For example, a future implementation of the Topo-Speech algorithm could convey dimensions such as depth through different manipulations such as volume and more or through other sensory stimuli, as suggested above. Another such “dimension” could be one of color—it is now known that the blind have a concept of color though historically thought to be “ungraspable” to those who have never experienced it (Kim et al., 2021) and that colors can affect spatial perception for dimensions and size (Oberfeld and Hecht, 2011; Yildirim et al., 2011). This dimension of perception, not currently available to the blind, could be conveyed similarly to the one used in the EyeMusic algorithm, developed in our lab by using different timbres of sound.

This study also provides a stepping stone toward fMRI studies of the sighted, the blind, and the visually impaired using the Topo-Speech algorithm. Aside from the activation of language areas such as Broca’s area and auditory areas, we would expect to see activation in the visual cortex of the blind. It would be of particular interest to compare the visual cortex activation in the blind to that in the visually impaired and the sighted when performing the task when blindfolded. Such a study could possibly shed light on the differences between the blind and the visually impaired concerning compensation by way of neuroplasticity and the extent thereof. In addition, we would be interested in seeing whether there are areas activated specifically for the combination between audition and language with respect to spatial perception.

When implemented in different systems, the Topo-speech algorithm could have several practical use cases representing two general categories, set and non-set scene implementations. Set scenes are ones where the information to be portrayed is fixed and not dynamically changing. An example of such scenarios would be using the Topo-Speech algorithm in a system that provides information to the blind/visually impaired concerning emergency exits in buildings, information about items in a museum, or elements in virtual reality situations. Systems designed for use in non-set scenes (in which objects and their locations change dynamically), could incorporate real-time artificial intelligence, for example, using image recognition to identify the objects to be named by the algorithm. The incorporation of artificial intelligence could open a wealth of possibilities for the blind and visually impaired with respect to providing them with freedom and independence in unfamiliar or changing environments. Artificial intelligence is already being integrated into rehabilitative systems for the sensory impaired, for example retinal prostheses (Barnes, 2012; Weiland et al., 2012) and hearing aids (Crowson et al., 2020; Lesica et al., 2021), yet SSD systems hold the potential for providing a more transparent and automatic perceptual experience (Ward and Meijer, 2010; Maimon et al., 2022) and therefore could be particularly powerful when combined with real time computer vision.

In addition, as the content represented by the algorithm can theoretically be adapted at will, one can imagine different operational modalities, that could even be alternated between by the user to match their needs, or automatically according to different use cases. For example, a previous study conducted by our institute has shown the feasibility of adding a “zooming in” element for increasing resolution when using a visual to auditory sensory substitution device in the blind (Buchs et al., 2016). An advanced implementation of the Topo-Speech could incorporate a “zooming in” feature to increase the resolution from “fruit” to “banana,” “apple,” or a “zooming out” feature to allow for general contextualization for example “home” or “gym.” These features can be particularly useful to the blind for independence and navigation in space. In addition, the algorithm could be attuned by the user to specific contextual categories, such as navigation elements (elevator, stairs) or people located spatially within a scene (supermarket crowded with people and objects), identification of the age or gender of people in different spatial locations of the scene, and more.

## Data availability statement

The original contributions presented in this study are included in the article/supplementary material, further inquiries can be directed to the corresponding author.

## Ethics statement

The studies involving human participants were reviewed and approved by the Reichman University Institutional Review Board (IRB). The patients/participants provided their written informed consent to participate in this study.

## Author contributions

AM: writing—original draft, writing—review and editing, methodology, conceptualization, visualization, project administration, and supervision. IW: writing—original draft, writing—review and editing, methodology, conceptualization, and visualization. MB: writing—original draft, investigation, and formal analysis. SC: writing—original draft, investigation, visualization, and formal analysis. ON and BH: methodology, conceptualization, and investigation. AA: writing—original draft and writing—review and editing, project administration, supervision, resources, conceptualization, investigation, methodology, funding acquisition. All authors contributed to the article and approved the submitted version.
